# Vitamin D supplementation during pregnancy: Updated meta-analysis on maternal outcomes^☆^^☆☆^

**DOI:** 10.1016/j.jsbmb.2016.02.008

**Published:** 2016-11

**Authors:** Cristina Palacios, Luz Maria De-Regil, Lia K. Lombardo, Juan Pablo Peña-Rosas

**Affiliations:** a Nutrition Program, Department of Human Development, Graduate School of Public Health, University of Puerto Rico, San Juan, Puerto Rico; b Research and Evaluation, Micronutrient Initiative, Ottawa, Canada; c Department of Epidemiology and Infectious diseases Research, William Beaumont Hospital, Royal Oak, MI, USA; d Evidence and Programme Guidance, Department of Nutrition for Health and Development, World Health Organization, Geneva, Switzerland

**Keywords:** Vitamin D, Supplements, Pregnant women, Gestational diabetes, Pre-eclampsia

## Abstract

**Background:**

Vitamin D deficiency is highly prevalent during pregnancy. It has been suggested that vitamin D supplementation during pregnancy may reduce the risk of adverse gestational outcomes.

**Objectives:**

To update a previous meta-analysis on the effects of oral vitamin D supplementation (alone or in combination with other vitamins and minerals) during pregnancy on maternal 25(OH)D levels and risk of developing pre-eclampsia, gestational diabetes, preterm birth, impaired glucose tolerance, caesarean section, gestational hypertension and other adverse conditions.

**Methods:**

We searched for randomized and quasi-randomized trials through the Cochrane Pregnancy and Childbirth Group’s Trials Register, the International Clinical Trials Registry Platform, the Networked Digital Library of Theses and Dissertations, and direct communications with relevant organizations. Assessments of inclusion criteria, extraction of data from included studies, and risk of bias’ assessments of the included studies were done independently by two review authors.

**Results:**

We included 15 trials, excluded 27 trials and 23 trials are still ongoing/unpublished. Data from seven trials with 868 women suggest that pregnant women supplemented with vitamin D had significantly higher 25(OH)D levels compared to controls (mean difference: 54.7 nmol/L; 95% CI 36.6, 72.9). Two trials found a lower risk of preeclampsia (8.9% versus 15.5%; average risk ratio 0.52; 95% CI 0.25, 1.05) and two other trials found no difference in the risk of gestational diabetes with vitamin D supplementation. Also, three trials found that supplementation with vitamin D plus calcium reduced the risk of pre-eclampsia (5% versus 9%; average risk ratio 0.51; 95% CI 0.32, 0.80).

**Conclusion:**

Supplementing pregnant women with vitamin D led to significantly higher levels of 25(OH)D at term compared to placebo/control but results were inconsistent. Vitamin D supplementation, with or without calcium, may be related to lower risk of preeclampsia but more studies are needed to confirm this.

## Highlights

• Supplementing pregnant women with vitamin D significantly increases 25(OH)D at term but results were inconsistent.
• It is unknown at this point what is the clinical significance of this finding but there is some indication that vitamin D supplementation, with or without calcium, may reduce the risk of pre-eclampsia.
• More studies are needed to confirm these results and to determine the effects of vitamin D supplementation on the risk of other maternal outcomes, such as gestational diabetes, impaired glucose tolerance, caesarean section, gestational hypertension, other adverse conditions and maternal death.

## 1 Introduction

Vitamin D deficiency during pregnancy may be a common health problem worldwide. A recent review including 17 studies in pregnant and lactating women (2 in America, 6 in Europe, 1 in Africa, 7 in Asia, 1 in Oceania) found the highest prevalence of vitamin D deficiency in pregnant women from Asia [48]. The prevalence of vitamin D deficiency (serum 25(OH)D levels <50 nmol/L or 20 ng/mL) was particularly high in India (60%), Turkey (50%), Pakistan (45%), and Kuwait (38–41%). This prevalence increases with seasonal variation, with a greater prevalence during the winter months compared to the summer months [46], [47]. Differences in latitude have also been shown to influence the concentration of vitamin D in a majority of pregnant women [56].

Vitamin D status during pregnancy has an important effect on the fetus as it completely relies on the maternal stores for its growth and development. During pregnancy, 1,25(OH)_2_D increases early during pregnancy and continues to increase until delivery [44]. This large increase in 1,25(OH)_2_D appears to be dependent on available 25(OH)D levels but independent on calcium metabolism, which is a unique feature of pregnancy that allows such high levels of 1,25(OH)_2_D [49]. Therefore, maintaining high enough levels of 25(OH)D are important to sustain the increased levels of 1,25(OH)_2_D during pregnancy. Such levels are still yet to be determined but studies have shown that maternal vitamin D status is associated with various health outcomes during pregnancy.

Maternal vitamin D deficiency in pregnancy has been associated with an increased risk of pre-eclampsia, a condition associated with an increase in maternal and perinatal morbidity and mortality [8], [9], [27], [34], [37], [67]. Two meta-analyses including eight [59] and 31 [1] studies found significantly higher risks pre-eclampsia in women with vitamin D deficiency. In addition, vitamin D deficiency in early pregnancy has been associated with elevated risk for gestational diabetes mellitus [21], [70], [1], [66] and with caesarean section [43], [54].

Some health organizations recommend vitamin D supplementation during pregnancy, ranging from 200 to 400 IU/d (5 to 10 μg/d) [11], [62]. These doses may not lead to optimal serum 25(OH)D levels during pregnancy. However, there is controversy regarding the 25(OH)D levels that are considered adequate or optimal for overall health and during pregnancy. The US Institute of Medicine has determined that levels greater than 50 nmol/L or 20 ng/mL are adequate based on the current studies available [22], although many investigators consider that optimal levels should be higher (greater than 75 nmol/L or 30 ng/mL) [16], [28]. It has been suggested that regular supplemental dose of vitamin D of 1000–1600 IU (25–40 μg/d) might be necessary to achieve and maintain what many considered to be optimal levels in the body [16]. However, the dose of vitamin D needed to have an effect during pregnancy is not clear.

Therefore, we aimed to systematically review the effects of vitamin D supplementation during pregnancy on maternal outcomes against no supplementation.

## 2 Methods

### 2.1 Types of studies

In this updated review, we included randomized trials of vitamin D supplementation during pregnancy in apparently healthy pregnant women. Detailed methods are published elsewhere [17]. Briefly, we included any vitamin D dose, duration or time of commencement of supplementation and any gestational or chronological age, parity (number of births) and number of fetuses. We included trials testing vitamin D alone or in combination with other micronutrients as long as the intervention and the control group were treated similarly. We excluded studies where vitamin D was provided by injection or studies assessing food fortification.

### 2.2 Types of interventions

We included trials examining any of the following comparisons: (1) oral vitamin D supplements alone versus no treatment/placebo (no vitamins or minerals); (2) oral vitamin D and calcium supplements versus no treatment/placebo (no vitamin or minerals); (3) oral vitamin D and calcium supplements versus oral calcium supplements (but no vitamin D); (4) oral vitamin D, calcium and other vitamins and minerals supplements versus oral calcium and other vitamins and minerals supplements (but no vitamin D); or (5) oral vitamin D and calcium and other vitamins and minerals supplements versus other oral vitamins and minerals supplements (but no vitamin D or calcium). Trials comparing different doses of vitamin D supplementation only were excluded.

### 2.3 Outcomes

The primary outcomes were: (1) Pre-eclampsia (as defined by trialists); (2) Gestational diabetes (as defined by trialists); (3) Vitamin D status at term (25OHD in nmol/L); and Adverse events (e.g. hypercalcaemia, kidney stones).

The secondary outcomes were: (1) Impaired glucose tolerance (as defined by trialists); (2) Caesarean section; (3) Gestational hypertension (as defined by trialists); and (4) Maternal death (death while pregnant or within 42 days of termination of pregnancy).

### 2.4 Search methods for identification of studies

The search methods were based on a standard template used by the Cochrane Pregnancy and Childbirth Group, as published earlier [17]. We searched the Cochrane Pregnancy and Childbirth Group’s Trials Register (23 February 2015), the World Health Organization (WHO) International Clinical Trials Registry Platform (31 January 2015), the Networked Digital Library of Theses and Dissertations (28 January 2015). For ongoing and unpublished studies, we contacted different institutions, such as the WHO (Reproductive Health and Research, Nutrition for Health and Development, and regional offices), UNICEF, the Micronutrient Initiative, the Global Alliance for Improved Nutrition and the US Centers for Disease Control and Prevention. We did not apply any date or language restrictions but we only found English language papers.

### 2.5 Selection of studies and data extraction

Two review authors (LL, JP) independently assessed for inclusion criteria. All the papers were assessed in duplicate and we resolved any disagreements through discussion or, if required, we consulted a third author (LMD). We contacted authors in the case of abstracts or studies with limited information. A data extraction form was used and data was entered into Review Manager Software [50] and checked for accuracy. Risk of bias was assessed in each study as previously published [17].

### 2.6 Statistical analyses

For dichotomous data, we present results as average risk ratio with 95% confidence intervals. For continuous data, we used the mean difference as the outcomes were measured in the same way between trials; there was no need to use the standardized mean difference to combine trials. For studies with more than two intervention groups (multi-arm studies), we combined groups to create a single pair-wise comparison [25] and included the disaggregated data in the corresponding subgroup category. When the control group was shared by two or more study arms, we divided the control group (events and total population) over the number of relevant subgroup categories to avoid double counting the participants.

For all outcomes, we carried out analyses on an intention-to-treat basis. The denominator for each outcome in each trial was the number randomized minus any participants whose outcomes are known to be missing. We assessed statistical heterogeneity in each meta-analysis using the Tau^2^, I^2^ and Chi^2^ statistics. We regarded heterogeneity as substantial if I^2^ was greater than 30% and either Tau^2^ was greater than zero, or there was a low P value (less than 0.10) in the Chi^2^ test for heterogeneity. All analyses were performed using the Review Manager software [50]. Since we detected substantial statistical heterogeneity, we used random-effects meta-analysis to produce an overall summary of an average treatment effect across trials. We treated the random-effects summary as the average range of possible treatment effects and we discussed the clinical implications of treatment effects differing between trials.

As only one study was considered of high quality, we did not conduct sensitivity analysis. We considered a study to be of high quality if it was assessed as having low risk of bias in both the randomization and allocation concealment and additionally a low risk of bias in either blinding or losses to follow-up. In addition, we were not able to conduct the subgroup analyses as very few studies contributed data. As more data become available, in updates of the review, we hope to explore possible subgroup differences by carrying out both visual exploration and formal statistical tests.

## 3 Results

### 3.1 Description of studies

**Fig. 1:**
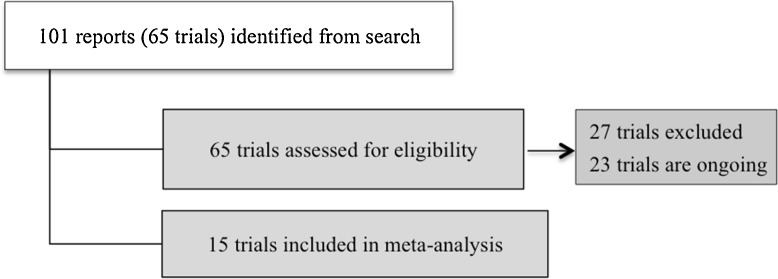
Study selection process.

A total of 15 studies involving 2833 women were included for this review (Fig. 1). We excluded 27 studies, mainly because the comparisons were among different doses of vitamin D without a placebo or control group [7[15], [24], [35], [40], [45], [51], [55], [57], [58], [29], [64], [65], [68]. Also, four trials were not randomised [2], [12], [14], [33] and three were done in pregnant women with chronic conditions [3], [4], [20]. Three trials were excluded for other various reasons [13], [31], [32], [36], [60], [63]. We identified 16 ongoing trials.

A detailed description of each included trial is shown in Table 1. Trials were conducted in Bangladesh [52], Brasil [19], China [34], France [18], [38], India [41], [39], [53], Iran [6], [5], [61], New Zealand [23], Russia [42] and the United Kingdom [10]. The seasons varied among studies with some trials occurring during the winter-spring period [18]; winter [38]; summer [69] or in different seasons [10], [19], [23], [53] but most did not report the season. The sample size from all the studies was small and ranged between 40 [18] and 400 women [41]. In all the studies women were recruited and received the supplements at 20 or more weeks of gestation. Pre-gestational body mass index was only reported in one trial and was used as a stratification criterion prior to the randomization [6], [5], [19], [53], [61]. Most trials did not specify the ethnicity or skin pigmentation of participants.

With respect to types of interventions (Table 1), nine trials compared provision of oral vitamin D supplement in comparison with placebo or no intervention [5], [10], [18], [23], [38], [39], [52], [53] and six trials compared oral vitamin D plus calcium supplements versus no treatment or placebo [34], [41], [42], [61]. The vitamin D dose used varied considerably. The daily doses ranged from 200 IU [6], [19], [34], [42], [61]; 400 IU [5], [34]; 800 IU [69], 1000 IU [10], [18], [23], [38]; 1200 IU [41] to 2000 IU [23]. Among the trials using large single doses, the dose varied from 50,000 IU [5]; 200,000 IU [69]; 60,000–480,000 IU [53]; 600,000 IU [39] to 35,000 IU vitamin D per week [52]. The type of vitamin D used in most trials was cholecalciferol-D3, with only three trials using ergocalciferol-D2 [10], [38], [69]. However, the form used was not reported in two of the trials [41], [39].

Different laboratory methods were used to measure vitamin D status as serum 25(OH)D levels (Table 1). Only two trials used mass spectrometry [23], [52]. The other trials used commercially available kits. However, four trials did not report the laboratory method used or if it was measured [69], [41], [39], [42].

### 3.2 Oral vitamin D alone supplements versus no treatment/placebo (no vitamins or minerals)

Nine studies involving 1251 women were included in this comparison [5], [10], [18], [23], [39], [52], [53], [69].

**Fig. 2:**
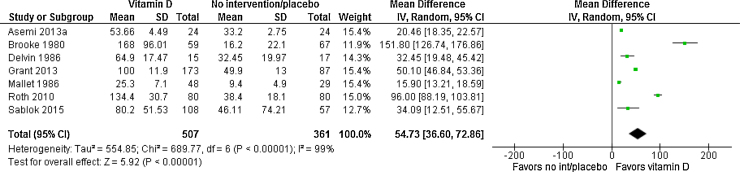
Forest plot of the effects of vitamin D supplementation on serum 25(OH)D levels.

With respect to maternal vitamin D levels at term, data from seven trials [5], [10], [18], [38], [52], [53], [69] with 868 women showed that vitamin D supplementation significantly increased 25(OH)D levels compared to placebo/control group, with a mean difference of 54.7 nmol/L (95% CI 36.6, 72.9) (Fig. 2). This response was highly heterogeneous (Tau^2^ = 216, I^2^ = 99% and Chi^2^ test for heterogeneity P < 0.00001). In addition, women who received vitamin supplementation on a daily basis reached a higher 25(OH)D levels at the end of the pregnancy compared with women who received a single dose (mean difference in 25(OH)D levels between groups of 37.7 nmol/L; 95% CI 28.3, 47.2). This response was also highly heterogeneous ((Tau^2^ = 554.9, I^2^ = 99% and Chi^2^ test for heterogeneity P < 0.00001).

With respect to preeclampsia, two trials [5], [53] with 219 women assessed this outcome. There was a trend for the effect of supplementation whereas women receiving vitamin D supplementation had a lower risk of pre-eclampsia than women on the placebo group (8.9% versus 15.5%; average risk ratio 0.52; 95% CI 0.25, 1.05). The risk of gestational diabetes was assessed in two trials [5], [53] with 219 women. Women taking vitamin D supplementation during pregnancy had similar risk than those on the placebo group (average risk ratio 0.43; 95% CI 0.05, 3.45).

With respect to adverse side effects, a single study including 135 women reported on this outcome [69], limiting our ability to assess the safety of the intervention. No trials reported on our other pre-specified maternal secondary outcomes: impaired glucose tolerance; caesarean section; gestational hypertension or maternal death.

### 3.3 Oral vitamin D and calcium supplements versus no treatment/placebo (no vitamin or minerals)

There were six trials with 1688 women for this comparison [6], [19], [34], [41], [42], [61]. There were no studies reporting on maternal 25(OH)D levels. The effects on pre-eclampsia were assessed in three trials [6], [41], [61] with 1114 women. Results showed that vitamin D supplementation significantly reduced the risk of preeclampsia in those receiving vitamin D and calcium supplementation combined compared to those on placebo/control groups (5% versus 9%; average risk ratio 0.51; 95% CI 0.32, 0.80). Only one study assessed the effects on gestational diabetes [5], [3]; therefore, no analysis was done. In addition, no trials reported on our other pre-specified maternal secondary outcomes.

## 4 Discussion

This article summarizes an ongoing update of a previous Cochrane review on vitamin D supplementation in pregnancy [17]). We now included 15 trials (2833 women), nine of which compared vitamin D alone versus no treatment or placebo while six trials provided vitamin D plus calcium in comparison with no treatment.

The main finding was that vitamin D supplementation during pregnancy significantly raised serum 25(OH)D levels at the end of pregnancy, particularly if supplementation was daily versus weekly, monthly or once. However, this response was highly heterogeneous. These inconsistencies could be related to the different doses used in the trials included and also in the difference in methods to assess serum 25(OH)D. This biomarker is difficult and complex, with high variability in results between methods used [27]. High performance liquid chromatography mass spectrometry is the best available method [26] but only one trial used this method. Therefore, results should be interpreted with caution.

We also found that vitamin D supplementation, with or without calcium, may reduce the risk of pre-eclampsia. Observational studies have also found that maternal vitamin D deficiency in pregnancy increases the risk of pre-eclampsia [59]. The mechanism explaining this is not well known. The pathogenesis of preeclampsia involves a number of biological processes that may be directly or indirectly affected by vitamin D, including immune dysfunction, placental implantation, abnormal angiogenesis, excessive inflammation, and hypertension [8], [9]. Also, in the only 2 studies reporting on gestational diabetes, no significant effect of vitamin D on this outcome was observed. Observational studies have reported an association between maternal vitamin D deficiency and gestational diabetes [21], [70], [1], [66]. Therefore, more studies are warranted.

The other maternal outcomes (impaired glucose tolerance, caesarean section, gestational hypertension, side effects or death) were either not reported or reported only by one trial; therefore, we were not able to assess the effects of the intervention. Observational studies have reported an association between maternal vitamin D deficiency and caesarean section [43], [54].

It is important to note that trials only comparing different doses of vitamin D supplementation were not included in this review. This was done to first evaluate the biological rationale for supplementing vitamin D during pregnancy against no supplementation on functional pregnant outcomes, as routine vitamin D supplementation is not widely done throughout the world. Once this is clearly determined, future reviews should evaluate the most optimal dose to use for significant beneficial effects on these pregnancy outcomes. In fact, Hollis et al. [29] and Hollis et al. [30] found significant beneficial effects of high doses of vitamin D supplementation (4000 IU/d) compared to lower dose (400 or 2000 IU/d) during pregnancy on pre-eclampsia and caesarean section. There are currently about 15 trials and several others ongoing that could contribute data for evaluating this.

In conclusion, further high quality rigorous randomized trials are required to evaluate the role of vitamin D supplementation in functional outcomes of pregnancy and its safety. Future research should evaluate if an increase of serum 25(OH)D levels improve maternal outcomes in populations with different degrees of body mass index, skin pigmentation and settings. Information on the most effective and safe dosage, supplementation regimen (daily, intermittent or single doses), the timing of initiation of vitamin D supplementation, and the effect of vitamin D when combined with other vitamins and minerals are also needed to inform policy-making. To our best knowledge there are currently 23 ongoing studies that, once published, are likely to double the body of evidence identified for this review. After their publication and overall assessment, conclusions on the effects and safety of this intervention may be updated.

## Conflict of interest

The authors have no conflict of interest to disclose.

## Contributions of authors

For this update Lia Lombardo and Juan Pablo Peña-Rosas assessed eligibility of the new trials and extracted the data in duplicate. Any differences were discussed and resolved with Luz Maria De-Regil and Cristina Palacios. Cristina Palacios prepared a draft of this manuscript with input from all authors.

## Declarations of interest

We certify that we have no affiliations with or involvement in any organization or entity with a direct financial interest in the subject matter of the review (e.g. employment, consultancy, stock ownership, honoraria, expert testimony).

Juan Pablo Peña-Rosas is full time staff of the World Health Organization. The authors alone are responsible for the views expressed in this document.

## Disclaimer

The authors alone are responsible for the views expressed in this article and they do not necessarily represent the views, decisions or policies of the institutions with which they are affiliated.

## Figures and Tables

**Table 1:** Description of included studies in the review.

Author	Country	Population	Intervention	Outcomes	Measurement of 25(OH)D
[6]	Republic of Iran (latitude: 35°44′N; North of the Tropic of Cancer).	54 primigravida singleton pregnant women at risk for pre-eclampsia, 18–35 years old, at their 3rd trimester.	RCT, single-blinded, with 2 arms: (1) Vitamin D3 (200 IU/d) + calcium (500 mg/d)(n = 27); or (2) Placebo (n = 27), followed for 9 weeks until term.	BMI, serum 25(OH)D levels, FBG, total cholesterol, TAG, HDL-c, LDL-c, dietary intakes, total HDL:cholesterol ratio, gestational diabetes, severe pre-eclampsia, preterm delivery.	Immunoassay (commercial ELISA kit)

[5]	Republic of Iran (latitude: 35°44′N; North of the Tropic of Cancer).	54 pregnant women 18–40 years old, with gestational diabetes mellitus (diagnosed by a 100-g oral OGTT at 24–28 wks gestation)	RCT, double-blinded, with 2 arms: (1) Vitamin D3 (50,000 IU) (n = 27); or (2) Placebo (n = 27), given at baseline and at day 21, followed for 6 weeks (Jan-April 2013). All participants were supplemented with 400 μg/d of folic acid and 60 mg/d of iron.	BMI, serum 25(OH)D levels, FBG; gestational diabetes mellitus; total glutathione; hs C-reactive protein; OGTT; QUICKY; total antioxidant capacity; placenta abruption, fetal death, severe preeclampsia.	Immunoassay (commercial ELISA kit)

[10]	London, England (latitude: 51°36′N; North of the Tropic of Cancer)	126 Asian immigrant pregnant women at 28–32 weeks of gestation.	RCT, double-blinded, with 2 arms: (1) Vitamin D2 (1000 IU/d) (n = 59); or (2) Placebo (n = 59), followed for 8–12 weeks until term.	Weight gain, dietary vitamin D intake, intervention compliance, serum 25(OH)D levels, plasma calcium, i-phosphate, bilirubin, albumin, alkaline phosphatase activity, vitamin D binding globulin and others.	Competitive protein binding assay

[18]	Lyon, France (latitude: 45°45′N; North of the Tropic of Cancer)	40 singleton pregnant women on their 3rd month of pregnancy	RCT, with 2 arms: (2) Vitamin D3 (1000 IU/d) (n = 20); or (2) No treatment (n = 20); followed for 12 weeks until term	Serum 25(OH)D levels, PTH, total calcium, ionised calcium, magnesium, inorganic phosphate.	Competitive protein binding assay

[19]	Rio de Janeiro, Brazil (latitude: 23°43′S; North of the Tropic of Capricorn).	84 primigravida, singleton pregnant adolescents (13–19 years) with 23–29 wks of gestation	RCT, with 2 arms: (1) Vitamin D3 (200 IU/d) + calcium (600 mg/d) (n = 43); or (2) Placebo (n = 41)	Serum 25(OH)D levels, PTH, IGF-I, lumbar spine PA, bone mineral content, serum prolactin and estradiol.	Immunoassay (Liaison; Diasorin)

[23]	Auckland, New Zealand (latitude: 36°52′S; South of the Tropic of Capricorn)	260 pregnant women 26–30 wks gestation, with a singleton pregnancy	RCT, double-blinded, with 3 arms: (1) Vitamin D3 (1000 IU/d) (n = 87); Vitamin D3 (2000 IU/d) (n = 86); or (3) Placebo (n = 87), from 26–30 wks of pregnancy until term (April 2010 to July 2011)	Serum 25(OH)D levels	LC–MS

Li 2000	Xi’an, China (latitude: 34°15′ N; North of the Tropic of Capricorn)	88 pregnant women at 20–24 weeks’ gestation and a BMI <24 kg/m^2^	RCT with 3 arms: (1) Vitamin D3 (200 IU/d) + calcium (600 mg/d) (n = 29); (2) Vitamin D3 (400 IU/d) + calcium (1200 mg/d) (n = 29); (3) No intervention (n = 30), from 20–24 weeks until delivery.	Blood pressure, ionized calcium and platelet intracellular calcium, incidence rates of pregnancy-induced hypertension.	Not reported
[38]	Northwest of France (latitude: 49°26′N; North of the Tropic of Cancer)	77 white pregnant women 18–36 years of age in the last trimester of pregnancy	RCT, double-blinded, with 3 arms: (1) Vitamin D2 (1000 IU/d for the last 3 months of pregnancy) (n = 21); (2) Vitamin D2 (single dose of 200,000 IU at the 7th month of pregnancy) (n = 27); or (3) No treatment (n = 29), followed until term	Serum and cord blood levels of 25(OH)D and 1,25(OH) 2D, 24-h urinary calcium excretion, serum calcium	Competitive protein binding assay

[41]	Rohtak, India (latitude: 76°34′N; North of the Tropic of Cancer)	400 pregnant women 20–35 years of age	RCT, with 2 arms: (1) Vitamin D (1200 IU/d) + calcium (375 mg/d) (n = 200); or (2) No supplementation (n = 200), followed from 20–24 wks of pregnancy until term	Pre-eclampsia; systolic and diastolic blood pressure at 24, 28, 32 and 36 weeks of gestation; serum calcium and creatinine.	Not assessed

[39]	Rohtak, India (latitude: 76°34′N; North of the Tropic of Cancer)	200 pregnant women, aged 22–35 years old, singleton	RCT, with 2 arms: (1) Vitamin D (2 doses of 600,000 IU at 7th and 8th month of pregnancy) (n = 100); or (2) No supplementation (n = 100), followed for 12 weeks until term	Serum calcium, proteins, i-phosphate, alkaline phosphatase, body weight; radiological examination; symptoms (back age, leg-pains, general weakness, cramps)	Not assessed

[42]	Moscow, Russia (Latitude: 55°45′N; North of the Tropic of Cancer).	72 pregnant women aged 18–35 with low calcium consumption ( < 600 mg/day)	RCT, with 2 arms: (1) Vitamin D3 (200 IU/d) + calcium (1250 mg/d) (n = 43); or (2) No intervention (n = 29), followed from 2nd trimester until term	Resistance of uterine arteries, resistance of umbilical arteries, uterine-placental circulation.	Not assessed

[52]	Dhaka, Bangladesh (latitude: 23°51′N; At the Tropic of Cancer)	160 pregnant women aged 18 <35 years old with gestational age of 26–29th week.	RCT, with 2 arms: (1) Vitamin D3 (35,000 IU/wks) (n = 80); or (2) Placebo (n = 80), followed from 26–29 weeks until term.	Serum 25(OH)D levels, serum calcium concentration, urine Ca:Cr ratio.	HPLC–MS
[53]	New Delhi, India (latitude: 28°38′N; North of the Tropic of Cancer)	180 pregnant women with singleton pregnancy at 14–20 week gestation.	RCT, with 2 arms: (1) no supplementation (n = 60); (2) Vitamin D3, which depended on initial level (single dose of 60,000 at 20 weeks, 120,000 at 20 and 24 weeks or 120,000 IU at 20, 24, 28 and 32 weeks).	Serum 25(OH)D, calcium, phosphorus and ALP levels; preterm labour, pre-eclampsia, gestational diabetes mellitus.	Sandwich ELISA
[61]	Isfahan, Iran (latitude: 33°52′N; North of the Tropic of Cancer)	990 nulliparous women with singleton pregnancy before 20 weeks of gestation and normal blood pressure	RCT, with 3 arms: (1) Aspirin (65 mg/d) (n = 330); (2) Vitamin D3 (200 IU/d) + calcium (500 mg/d) (n = 330); (3) no intervention (n = 330), from week 20 until delivery.	Blood pressure, weight, height, BMI, urine protein, duration of gestation	Not assessed

[69]	London, England (latitude: 51°36′N; North of the Tropic of Cancer)	180 pregnant women (from diverse ethnicity/race) at 27 wks gestation	RCT with a 4 × 3 block design: (1) Vitamin D2 (800 IU/d)(n = 60); (2) Vitamin D3 (single dose of 200,000 IU) (n = 60); or (3) No supplementation (n = 60), followed for 13 weeks until term (April-Nov 2007)	Serum and cord 25(OH)D levels at delivery, PTH and corrected calcium levels at delivery, adverse events	Not specified

RCT: randomized controlled trial; BMI: body mass index; TAG: triacylglyceride; FBG: fasting blood glucose; OGTT: oral glucose tolerance test; QUICKY: quantitative insulin sensitivity check index; PTH: parathyroid hormone; IGF: insulin growth factor; HDL-c: high density lipoprotein cholesterol; LDL-c: low density lipoprotein cholesterol. LC–MS: Liquid chromatography–mass spectrometry; HPLC–MS: high-Performance liquid chromatography-mass spectrometry.
